# PANDAseq: paired-end assembler for illumina sequences

**DOI:** 10.1186/1471-2105-13-31

**Published:** 2012-02-14

**Authors:** Andre P Masella, Andrea K Bartram, Jakub M Truszkowski, Daniel G Brown, Josh D Neufeld

**Affiliations:** 1 Department of Biology, University of Waterloo, Waterloo, Ontario, Canada; 2 David R. Cheriton School of Computer Science, University of Waterloo, Waterloo, Ontario, Canada

## Abstract

**Background:**

Illumina paired-end reads are used to analyse microbial communities by targeting amplicons of the 16S rRNA gene. Publicly available tools are needed to assemble overlapping paired-end reads while correcting mismatches and uncalled bases; many errors could be corrected to obtain higher sequence yields using quality information.

**Results:**

PANDAseq assembles paired-end reads rapidly and with the correction of most errors. Uncertain error corrections come from reads with many low-quality bases identified by upstream processing. Benchmarks were done using real error masks on simulated data, a pure source template, and a pooled template of genomic DNA from known organisms. PANDAseq assembled reads more rapidly and with reduced error incorporation compared to alternative methods.

**Conclusions:**

PANDAseq rapidly assembles sequences and scales to billions of paired-end reads. Assembly of control libraries showed a 4-50% increase in the number of assembled sequences over naïve assembly with negligible loss of "good" sequence.

## Background

Single-gene sequencing has become the benchmark for studying microbial taxonomic composition of environmental samples, by amplification of hypervariable regions of the 16S rRNA gene. Next-generation sequencing platforms, such as Illumina, are now adapted for the generation of multi-million-member sequence libraries for sample comparisons [1-4]. The PCR amplicons used for sequencing typically encompass one or more 16S rRNA gene hypervariable regions and amplicon lengths typically extend beyond the sequencing limit of the Illumina single-read method, which is typically less than 150 bases. Because the Illumina platform can generate amplicon sequences in a paired-end format, based on each template's position on the flow cell, paired reads can be directly matched and assembled. The prefiltering step of the genome assembly software PHRAP can be used to assemble reads [3]. Although the Needleman-Wunsch algorithm [5] embedded in Merger (http://emboss.sourceforge.net/apps/release/6.2/emboss/apps/merger.html) has been used to assemble Illumina paired-end reads [6], PANDAseq makes use of Illumina-specific properties, including the low probability of gap-inclusion.

**Figure 1:**
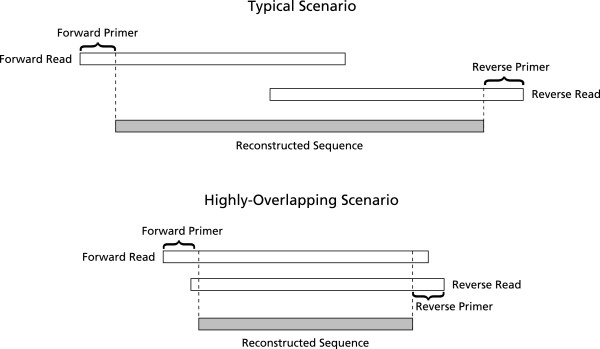
**Schematic of paired-end assembly**. Typical scenario: forward and reverse reads are overlapped and the primer regions are removed to reconstruct the sequences. Highly overlapping scenario: for short templates, the overlapping region may include the primer regions.

Assembly of the Illumina paired-end sequences can be done naïvely requiring perfect match in the region of overlap, to produce large numbers of correct sequences, as in the first iteration of our assembly software [1]. However, approximately 40% of the sequences were discarded due to uncalled or miscalled bases. The proportion of discarded paired-end reads, due to bases uncalled or miscalled, will increase as read lengths increase, decreasing naïve assembly effectiveness. We suggest a more sophisticated method that corrects errors probabilistically with the overlap data from the paired-end reads. When the overlap between the forward and reverse reads is substantial, many uncalled or miscalled bases can be corrected using the complementary sequence. Our software, PANDAseq, uses paired-end Illumina reads, determines the proper amount of overlap and reconstructs the entire sequence by correcting errors in the overlapping region (Figure 1). Assembly is extremely fast and millions of paired-end reads can be rapidly assembled on a desktop computer.

## Implementation

PANDAseq aligns each set of paired-end sequence reads in a three-step process. First, it determines the locations of the amplification primers, if they are specified and were sequenced. Then, it identifies the optimal overlap. Finally, it reconstructs the complete sequence, correcting any errors, and checks for various constraints, such as length and quality.

To score alignments, we calculate the probability that the true nucleotides,  and , are the same, given the observed nucleotides, *X *and *Y*. We estimate this with the included quality information found in the Illumina reads. For each base, CASAVA provides an encoded quality score, which is the probability of the base being miscalled. This probability (*ϵ*) is approximated by $10^{-\frac{A_{0}-64}{10}}=10^{-\frac{A_{1}-33}{10}}$ where *A*_0 _is the ASCII quality value in the Illumina analysis pipeline versions before CASAVA 1.8 and *A*_*1 *_is the ASCII value used in CASAVA 1.8. [7]

Assuming all nucleotides are equally likely (i.e., the prior probability that the true bases match is $\frac{1}{4}$), and that sequencing errors are independent and result in equiprobable choices over the other three nucleotides, the probability that the true bases match, given that the sequenced bases match, is:

$$\text{Pr}\left[\hat{X}=Ŷ|X=Y\right]=\left(1-\varepsilon_{X}\right)\left(1-\varepsilon_{Y}\right)+\frac{\varepsilon_{X}\varepsilon_{Y}}{3}$$

and the probability that the true bases match, given the sequenced bases mismatch, is:

$$\begin{array}{ll} \text{Pr}\left[\hat{X}=Ŷ|X\neq Y\right] & =\;\frac{1}{3}\left(1-\varepsilon_{X}\right)\varepsilon_{Y}\;\\ & \;+\frac{1}{3}\left(1-\varepsilon_{Y}\right)\varepsilon_{X}\;\\ & \;+\frac{2}{9}\varepsilon_{X}\varepsilon_{Y}\;\\ & \; \end{array}$$

If one of the bases is an uncalled base, N, then the probability that the bases match is:

$$\text{Pr}\left[\hat{X}=Ŷ|Y=N\right]=\frac{1}{4}$$

Using these probabilities, PANDAseq begins the assembly process by determining the positions of forward and reverse primers, if supplied. To accomplish this, the program finds the first offset, *x*, where the primer aligns. For a primer *P *and a sequence *S*, the program calculates

$$\overset{\left|P\right|-1}{\underset{i=0}{\prod }}\text{Pr}\left[{Ŝ}_{i+x}=P_{i}\right]$$

while assuming that $\varepsilon_{P_{i}}=1-10^{-4.1}$, which is the highest value score assigned by Illumina [8] and, intuitively, assuming that  is *P.*

The program then finds the best overlap greater than a specified threshold for the forward and reverse sequences, *F *and *R*, respectively. If no suitable overlap is found, then the read pair is discarded. This is done for the entire read, even if there are primers to be removed, as it is possible for the overlap to be sufficiently long to be in the primer region. A schematic is shown in Figure 1.

The value of *c *∈ [1, min(|*F*|, |*R*|)) is chosen which maximises this formula:

$$\begin{array}{ll} \text{Pr}\left[F,R|c\right] & =\;\underset{i=1\ldots f}{\prod }\text{Pr}\left[F_{i}\right]\;\\ & \;\cdot \underset{i=1\ldots c}{\prod }\text{Pr}\left[{\hat{F}}_{i+f}={\hat{R}}_{i}\right]\;\\ & \;\cdot \underset{i=1\ldots r}{\prod }\text{Pr}\left[R_{i+c}\right]\;\\ & \; \end{array}$$

where $\text{Pr}\left[F_{i}\right]=\frac{1}{4}$ and $\text{Pr}\left[R_{i}\right]=\frac{1}{4}$ and the remainder is as above with e fixed at a value determined empirically to be the average error rate. This value of *ϵ *was calculated by counting the mismatch rate in known index tags in a defined community data set (described below). This parameter need not be retuned as it is only an estimate of the error. Because the index read is short and sequenced earlier in the process, it likely has fewer errors and, therefore, its error rate should underestimate the true error rate. Regardless, the error rate specified for this step should not negatively affect the ability of PANDAseq to identify the best overlap for the forward and reverse reads.

Once the overlap is selected, the output sequence is constructed and an overall quality score is calculated. During this process, the primer regions are disregarded if primers were specified. The unpaired regions are copied from the available strands and the quality score for these regions is the product of the probability of those bases being correct. For the overlapping region, the decision-making process is more complex. If the bases agree, the base is included and the quality of this base is assumed to be $\text{Pr}\left[\hat{X}=Ŷ|X=Y\right]$. If the bases disagree, the base with the higher quality score is chosen and the quality of this base is assumed to be $\text{Pr}\left[\hat{X}=Ŷ|X\neq Y\right]$. If either or both bases are uncalled, they are considered to always match, noting that unassigned bases are always associated with the lowest quality score by CASAVA [8].

In certain cases, the CASAVA pipeline masks the quality score at the end of the read, replacing all quality scores with the lowest quality score [8]. In this case, special quality scoring is used by PANDAseq. If one base is masked, the probability of the other base is used if the bases match or uniformly random, $\frac{1}{4}$, is used if they do not match. If both are in the masked region, the quality is assumed to be uniformly random, $\frac{1}{4}$.

The constructed sequence can then be validated against user-specified criteria. The quality score assigned to the assembled sequence is the geometric mean of the quality scores calculated above, which compensates for the variable lengths of the final sequences. PANDAseq enables users to reject sequences based on low quality score, lengths that are too short or too long, or the presence of uncalled bases. A module system is also available within PANDAseq to allow more sophisticated validation of user sequences, such as verification of known secondary structure or conserved regions. Note that there is a detailed manual included with the software that describes example usage scenarios.

## Results and discussion

To validate PANDAseq, we used three experimental tests: (1) a test using simulated data to verify algorithmic correctness, (2) a test using sequence data from a single-template PCR amplicon to verify the quality of assembled reads, and (3) a test with experimental data obtained from a defined mixture of genomic DNA fragments to compare PANDAseq assembly yields with naïve assembly.

**Figure 2:**
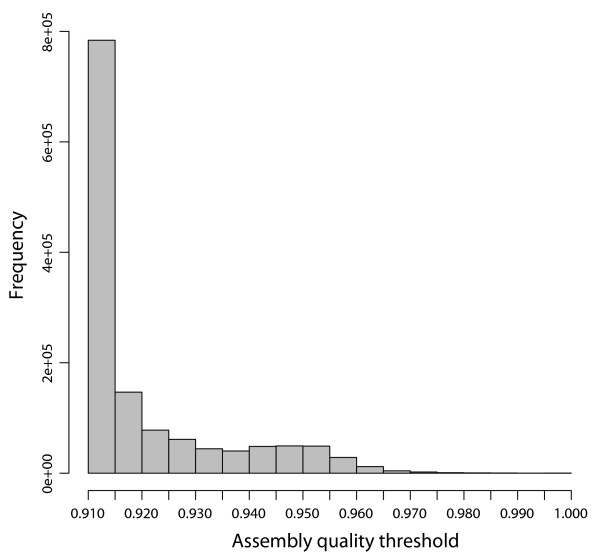
**Quality scores of assembled masked data**. A perfect 16S rRNA sequence from *Sinorhizobium meliloti *was masked using real Illumina quality scores and the resulting paired-end sequences were assembled with PANDAseq. A histogram of quality scores for the assembled sequences is shown.

Simulated data was useful in determining how real quality scores affect sequence assembly. We used a previously published Illumina sequencing run of V3 hypervariable regions from a defined library (described below) [1] and replaced the sequence with the corresponding region from *Sinorhizobium meliloti *(135 bases, region amplified by 341f and 518r excluding primers [9]), up to the length of the original reads. Although this V3 sequence was taken from the published genome, it corresponds to the region being sequenced in the experimental data such that any sequencing quality problems due to secondary structure are preserved. This provides simulated error-free reads with experimental quality scores. Though the assembly was then performed without a quality filter, all 1 350 602 synthesized paired-end sequences assembled with quality scores greater than 0.9 (Figure 2). This value establishes an upper limit on the quality score independent of sequencing errors; that is, setting the quality threshold higher than 0.9 would demand that reads have fewer errors than data known to be perfectly correct and is, in effect, demanding the underlying read quality be better than is necessary to reconstruct the sequence.

Further analysis was performed on a library constructed from a *Methylococcus capsulatus *(ATCC 33009) full length 16S rRNA gene amplification products cloned into the TOPO vector using the TOPO TA cloning kit (Invitrogen). The resulting construct was used as template for 16S rRNA gene amplification and sequencing on an Illumina GAIIx as previously described [1], with the exception of the PCR product gel excision and purification step, which used the Wizard SV Gel and PCR Clean-Up System (Promega). Sequencing produced 673 845 paired-end 108-base reads, available at http://neufeldserver.uwaterloo.ca/~apmasell/pandaseq_sampledata.tar. Of these, 598 775 sequences were assembled with an assembly quality score greater than or equal to 0.9. We assembled the same single-template data with a quality threshold of 0.6 and this increased the number of sequences assembled by 9%, yielding 652 249 sequences. The errors in the original, individual reads and the reconstructed sequences were counted and error information is shown in Table 1. Only two reads contained uncalled bases and were excluded. PANDAseq improved the correctness of the reconstructed sequence relative to the original reads or preserved the correctness of good reads. Depending on the quality threshold, only about 0.02-0.08% of output read contained errors introduced by the PANDAseq assembly process, as calculated from the results in Table 1; these introduced errors were substantially less than the 5-8% of sequences with errors corrected by PANDAseq. Given an assembly threshold of 0.9 as an upper limit, we then attempted to determine the lower limit for the quality threshold by looking at a comparable quality score of the unassembled reads. We determined the geometric mean of the read qualities of the sequences which assembled to be no lower than 0.7. Only 0.04% of reads had a quality score between 0.6 and 0.7. Therefore, if a sequence assembles, it is probably correct, given the quality of the underlying read, regardless of quality score.

**Table 1:** Read error correction frequencies

Quality (Geometric Mean)	0.9 - 1.0	0.6 - 0.9
Error-free Input and Output	544 669	21 095
All Errors Retained	4 023	4 675
Input Errors Reduced	50 082	27 668
Errors Introduced	0	37

Total	598 774	53 475

Summary of error frequencies in assembled Illumina paired-end reads generated from sequenced V3-region amplicons of *Methylococcus capsulatus *strain Bath.

All error data were analyzed solely within the region of overlap, which was relevant to PANDAseq assembly. Low-abundance "contamination" was observed in the dataset (data not shown), possibly due to reagents used for PCR. These will contribute to the counts of sequences that had errors that were retained. This category will also contain sequences in which both reads contain low-quality bases with quality scores masked by CASAVA.

**Figure 3:**
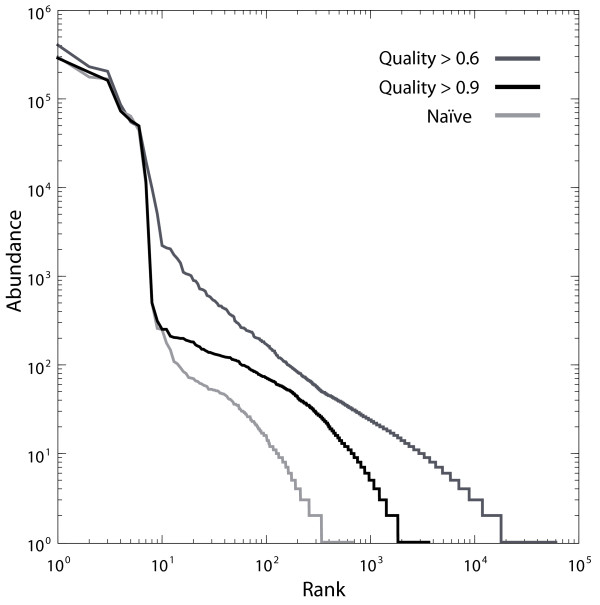
**Comparison of output of various assemblers**. A scatter plot of the percentage of paired-end sequence assemblies from sequenced V3-region amplicons of *Methylococcus capsulatus *strain Bath against the average number of mismatching nucleotides between the assembled sequence and the reference sequence. The comparison was done between PANDAseq and three alternative assemblers (see text).

We compared the quality of PANDAseq assembly against the existing assemblers: SHERA [10], iTags (using PHRAP) [3], and BIPES (using Merger) [6]. For this *M. capsulatus *library assembly, reads used still contained primers; primer removal was not a preprocessing step. If assembling sequences where the overlap region is large, it is possible that the end of one read would overlap the primer region of the other (see the highlyoverlapping scenario shown in Figure 1). PANDAseq assembled all sequences within 2 minutes 25 seconds, which is much faster than the second fastest method tested, SHERA, at 73 minutes. The other two programs were at least 100 times slower than PANDAseq. We were unable to use Merger on our test environment, a Macintosh Pro with 2 quad-core Intel Xeon 2.93 GHz processors, and so the BIPES assembler was run on a Linux machine with a quad-core Intel i5 3.2 GHz processor. Shown in Figure 3 is a scatter plot of accuracy versus coverage for the four different methods we considered. PANDAseq assembles the fewest reads in the dataset, but was, by far, the most accurate. iTags/PHRAP was inferior in coverage and accuracy to BIPES/Merger and to SHERA (Figure 3). Comparing average errors in the output sequence, PANDAseq performed the best with 0.38 nucleotide errors per sequence as compared to a minimum of 1.08 errors per sequence for SHERA and BIPES. However, as mentioned previously, PANDAseq assembled the fewest sequences at 95.5% of the dataset. SHERA assembled all sequences in the dataset, but it is worth noting that, upon inspection, many of the products assembled exclusively by SHERA were incorrect as an erroneous overlap region had been selected (data not shown). The number of error-free sequences in the overlap region is shown in Table 2. While SHERA has a larger number of sequences with correct overlaps, these represent a smaller fraction of the output compared to PANDAseq. Many of these sequences produced by SHERA with correct overlaps were rejected by PANDAseq due to low quality scores.

**Table 2:** Number of sequences with correct overlap regions

Assember	Correct Assemblies	Percentage of Output
PANDAseq	628 131	96.30
BIPES (Merger)	621 357	94.63
SHERA	637 646	92.35
iTags (PHRAP)	3 578	0.55

Summary of the number of correct overlap sequences in assembled output from sequenced V3-region ampli-cons of *Methylococcus capsulatus *strain Bath.

The percentage of sequences with error-free overlap regions is shown as a fraction of the total output for each assembler.

**Figure 4:**
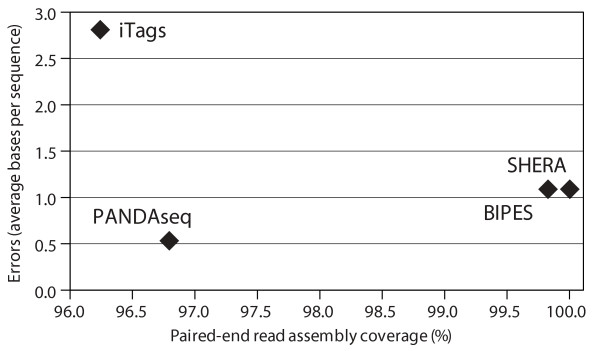
**Rank abundance curves for control libraries**. Rank-abundance curves for defined multi-organism libraries [1] assembled at two different quality thresholds using PANDAseq and naïve assembly followed by clustering with CD-HIT into OTUs of 97% identity.

Finally, we used a composite of previously published duplicate control library [1], made from mixed pure bacterial cultures (NCBI SRA accession SRA024100), to compare naïve assembly and PANDAseq. In this composite library, the most abundant sequences are from the added pure cultures, but there are other contaminant sequences, likely from the growth media used [1]. We performed the naïve assembly with the software used previously [1] and the PANDAseq assembler, discarding any sequences with uncalled bases. The assembled sequences were clustered at 97% identity using CD-HIT [11] and abundance curves were generated from the resulting clustered operational taxonomic units (OTUs; Figure 4). At a threshold of 0.9, the number of sequences increased 3.9% in total over naıve assembly, yielding an average increase of 2.1% in the most abundant clusters. There were 83 OTUs in which PANDAseq had fewer sequences than naïve assembly: 71 of them were OTUs for which the naïve assembly found a single sequence, while the PANDAseq assembly found zero. Relaxing the quality threshold increased sequence recoveries substantially. When the quality threshold was reduced to 0.6, the total number of sequences increased by 50% and the number of sequences in the most abundant clusters increased by 85%. Even if the quality threshold was lowered below 0.6, no new OTU sequences were assembled by PANDAseq. New, low-abundance OTUs were formed from some of the additional sequences, which, although they do not match the pure-culture organisms, classify taxonomically using the RDP classifier [12,13] (data not shown).

Assembling 1350 602 reads took just 364 seconds on a Macintosh Pro with 2 quad-core Intel Xeon 2.93 GHz processors. Profiling indicated that cache faults are the limiting factor in performance (data not shown), and the current design minimises cache faults during analysis of each sequence pair.

There is a concern that, when making a choice between two disagreeing bases, the reconstructed sequence does not reflect the true sequence. For the control library, the disagreeing bases were dominated by mismatches in the quality-masked region of the reads, where both bases were of low quality and the decision would be arbitrary because there is no reasonable way to discern which base is better. In those cases, the entire reads are of low quality and likely to be discarded due to the quality threshold. However, mismatches generally occur between a base with a high quality score and a base with a low quality score, simplifying the choice of which base is correct. In control library data, only 20% of mismatched bases both had quality scores masked by CASAVA. Since the quality masked region must be quite long for this to occur, only few sequences suffer strongly from these mismatched quality-masked bases. This is due to the overlap region typically being longer than the quality-masked regions.

## Conclusions

PANDAseq produces additional high-quality assemblies from Illumina paired-end reads than naïve assembly for minimal computational cost and provided more rapid and higher quality results compared to existing assemblers. Error correction, particularly of uncalled bases, increases the number of assembled sequences. Although it is possible for PANDAseq to produce incorrect assemblies, most assemblies are correct because incorrect assemblies have low quality scores, as these mismatches occur in quality-masked regions of both reads, and are discarded. This software provides a versatile and powerful way to assemble paired-end Illumina reads without otherwise discarding high-quality sequence data.

## Availability and Requirements

**Project name**: PANDAseq

**Project home page**: https://github.com/neufeld/pandaseq

**Operating system(s)**: POSIX-compliant (Windows, Linux, and MacOS)

**Programming language**: C

**Other requirements**: None

**License**: GNU GPL

**Any restrictions to use by non-academics**:

None

## Authors' contributions

The program requirements and experimental framework were developed by JDN and APM. The PANDAseq software was written by APM. The libraries used were constructed by AKB. Analysis and development of the error models was shared by JMT and DGB. All authors have read and approved the final version of the manuscript.
